# A Suppressor Mutation in the β-Subunit Kis1 Restores Functionality of the SNF1 Complex in *Candida albicans snf4*Δ Mutants

**DOI:** 10.1128/msphere.00929-21

**Published:** 2021-12-15

**Authors:** Bernardo Ramírez-Zavala, Austin Mottola, Ines Krüger, Joachim Morschhäuser

**Affiliations:** a Institute for Molecular Infection Biology, University of Würzburg, Würzburg, Germany; University of Georgia

**Keywords:** AMP-activated kinases, *Candida albicans*, Mig1, Mig2, SNF1

## Abstract

The heterotrimeric protein kinase SNF1 is a key regulator of metabolic adaptation in the pathogenic yeast Candida albicans, and mutants with a defective SNF1 complex cannot grow on carbon sources other than glucose. We identified a novel type of suppressor mutation in the β-subunit Kis1 that rescued the growth defects of cells lacking the regulatory γ-subunit Snf4 of the SNF1 complex. Unlike wild-type Kis1, the mutated Kis1^A396T^ could bind to the catalytic α-subunit Snf1 in the absence of Snf4. Binding of Kis1^A396T^ did not enhance phosphorylation of Snf1 by the upstream activating kinase Sak1, which is impaired in *snf4*Δ mutants. Nevertheless, the mutated Kis1^A396T^ reestablished SNF1-dependent gene expression, confirming that SNF1 functionality was restored. The repressor proteins Mig1 and Mig2 were phosphorylated even in the absence of Snf1, but their phosphorylation patterns were altered, indicating that SNF1 regulates Mig1 and Mig2 activity indirectly. In contrast to wild-type cells, mutants lacking Snf4 were unable to reduce the amounts of Mig1 and Mig2 when grown on alternative carbon sources, and this deficiency was also remediated by the mutated Kis1^A396T^. These results provide novel insights into the regulation of SNF1 and the repressors Mig1 and Mig2 in the metabolic adaptation of C. albicans.

**IMPORTANCE** The highly conserved protein kinase SNF1 plays a key role in the metabolic adaptation of the pathogenic yeast Candida albicans, but it is not clear how it regulates its downstream targets in this fungus. We show that the repressor proteins Mig1 and Mig2 are phosphorylated also in cells lacking the catalytic α-subunit Snf1 of the SNF1 complex, but the amounts of both proteins were reduced in wild-type cells when glucose was replaced by alternative carbon sources, pointing to an indirect mechanism of regulation. Mutants lacking the regulatory γ-subunit Snf4 of the SNF1 complex, which cannot grow on alternative carbon sources, were unable to downregulate Mig1 and Mig2 levels. We identified a novel type of suppressor mutation, an amino acid substitution in the β-subunit Kis1, which enabled Kis1 to bind to Snf1 in the absence of Snf4, thereby restoring Mig1 and Mig2 downregulation, SNF1-dependent gene expression, and growth on alternative carbon sources. These results provide new insights into the SNF1 signaling pathway in C. albicans.

## INTRODUCTION

The heterotrimeric protein kinase SNF1, a member of the highly conserved AMP-activated protein kinase family, has a key role in the metabolic adaptation of eukaryotic cells, especially when the preferred carbon source glucose becomes limiting and alternative carbon sources have to be utilized (1). In the model yeast Saccharomyces cerevisiae, the SNF1 complex consists of the catalytic α-subunit Snf1, the γ-subunit Snf4, and one of three alternative β-subunits—Gal83, Sip1, or Sip2 (the designations SNF1 and Snf1 refer to the heterotrimeric complex and the catalytic α-subunit, respectively [1]). Snf4 binds to the C-terminal regulatory domain of Snf1 and releases the N-terminal catalytic domain from autoinhibition under inducing conditions (2). The β-subunits, which also bind to a C-terminal region in Snf1, regulate the subcellular localization of the kinase and its interaction with target proteins (3–5). Snf1 is activated in response to glucose limitation and other stresses via phosphorylation at Thr210 in its activation loop by the three partially redundant upstream activating kinases Elm1, Sak1, and Tos3 (6–8). It is dephosphorylated at Thr210 and thereby inactivated by the Reg1-Glc7 protein phosphatase 1 (9, 10). To enable adaptation to glucose limitation, SNF1 adjusts the transcriptional program and the activity of metabolic enzymes and nutrient transporters (1). An important target of SNF1 in the regulation of gene expression is the repressor protein Mig1, which together with the functionally related repressor Mig2 inhibits the transcription of genes that are required for the utilization of alternative carbon sources (11–15). Phosphorylation by Snf1 under glucose-limiting conditions results in export of Mig1 from the nucleus and derepression of its target genes (13, 15, 16). In contrast, Mig2 inactivation in response to glucose limitation was found to be independent of Snf1, and Mig2 remained in the nucleus under these conditions (17). However, later studies demonstrated that Mig2 is phosphorylated in an Snf1-dependent fashion under other stress conditions that activate SNF1, resulting in its degradation (18) or export from the nucleus (19, 20).

In the pathogenic yeast Candida albicans SNF1 is also important for metabolic adaptation, and it is essential for the successful colonization of a mammalian host (21). The C. albicans SNF1 complex consists of the α-subunit Snf1, the γ-subunit Snf4, and one of the two β-subunits Kis1 and Kis2; Snf1 is constitutively phosphorylated by a single upstream activating kinase, Sak1 (21–23). The catalytic subunit Snf1 was thought to be essential for the viability of C. albicans, because no deletion mutants were obtained in several attempts by different research groups, but it was recently shown that *snf1*Δ mutants are viable and can grow under optimal conditions (21, 24–26). How SNF1 controls gene expression in C. albicans also has remained an open question for a long time, because Mig1 of C. albicans lacks the consensus Snf1 phosphorylation sites (27). However, it was recently demonstrated that deletion of *MIG1* mitigates the growth defects of *snf1*Δ and *snf4*Δ mutants, and the growth of *snf4*Δ mutants was further improved when both *MIG1* and *MIG2* were deleted (25, 28). Furthermore, deletion of *MIG1* and *MIG2* reversed the gene expression changes seen in a *sak1*Δ mutant lacking the SNF1-activating kinase (28). These observations indicate that Mig1 and Mig2 are directly or indirectly inhibited by SNF1 also in C. albicans.

In order to gain insight into the SNF1 signaling pathway in C. albicans, we had previously isolated spontaneous mutants of an *snf4*Δ strain that regained the ability to utilize sucrose as carbon source (29). As we report here, one of these mutants contained a missense mutation in *KIS1*, and we show that the mutated version of the γ-subunit Kis1 restores the functionality of SNF1 in the absence of its β-subunit Snf4, resulting in downregulation of the repressors Mig1 and Mig2 and reconstituted gene expression.

## RESULTS

### An A396T amino acid substitution in Kis1 suppresses *snf4*Δ mutant phenotypes.

**FIG 1:**
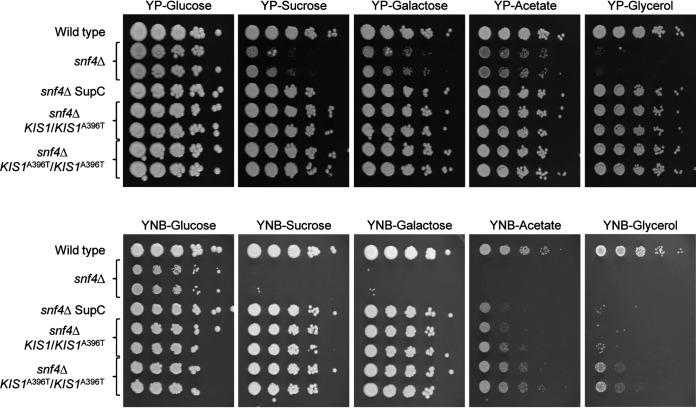
Growth of the wild-type strain SC5314, *snf4*Δ mutants, the spontaneous *snf4*Δ suppressor mutant (*snf4*Δ SupC), and derivatives of the *snf4*Δ mutants in which the suppressor mutation was introduced into one or both endogenous *KIS1* alleles on different carbon sources. YPD overnight cultures of the strains were adjusted to an OD_600_ of 2.0, and serial 10-fold dilutions were spotted on YP or YNB agar plates containing 2% glucose, sucrose, galactose, acetate, or glycerol as the carbon source. Plates were incubated for 2 (top panels) or 4 (bottom panels) days at 30°C. Both independently generated series of mutants are shown.

In a previous study, we had isolated two suppressor mutants that restored the ability of a C. albicans *snf4*Δ mutant to grow on sucrose as the main carbon source (29). One of the suppressor mutants, SCΔ*snf4*SupB, contained an in-frame deletion in one of the *SNF1* alleles, encoding the catalytic α-subunit Snf1 of the SNF1 complex, which restored Snf1 function in the absence of the regulatory γ-subunit Snf4 (29). The other suppressor mutant, SCΔ*snf4*SupC, did not contain a mutation in *SNF1* and the basis of the suppressor phenotype remained unknown. Since it was recently observed that deletion of the repressor-encoding genes *MIG1* and/or *MIG2* overrides growth defects of *snf1*Δ and *snf4*Δ mutants (25, 28), we determined the sequences of these genes in SCΔ*snf4*SupC. However, no mutations were detected in the coding regions of *MIG1* and *MIG2* compared to those of the wild-type parental strain SC5314. We hypothesized that an alteration in one of the β-subunits Kis1 or Kis2 might also relieve the dependence of Snf1 activity on the presence of the γ-subunit Snf4. Indeed, we found that SCΔ*snf4*SupC contained a G-to-A substitution at position +1186 in one of the *KIS1* alleles, which would result in an alanine-threonine exchange at amino acid position 396 of Kis1. To test whether this mutation was responsible for the suppressor phenotype, we introduced it into one of the *KIS1* alleles of the two independently generated *snf4*Δ mutants SCSNF1M4A and SCSNF1M4B and tested the growth of the resulting heterozygous strains on different carbon sources. As can be seen in Fig. 1, the engineered strains containing one copy of the *KIS1*^A396T^ allele grew as well as the original suppressor mutant, although their growth was still reduced compared to the wild-type strain SC5314, especially on minimal (YNB) media containing acetate or glycerol as carbon source. To examine whether the growth of *snf4*Δ mutants would be further improved if they contained only the mutated form of Kis1, we replaced also the remaining second wild-type *KIS1* allele by the *KIS1*^A396T^ allele in the *snf4*Δ mutants. Indeed, homozygosity for the mutated *KIS1* allele further improved the growth of *snf4*Δ mutants on minimal plates containing acetate or glycerol as the sole carbon source (Fig. 1).

### The A396T mutation restores binding of Kis1 to Snf1 in the absence of Snf4.

**FIG 2:**
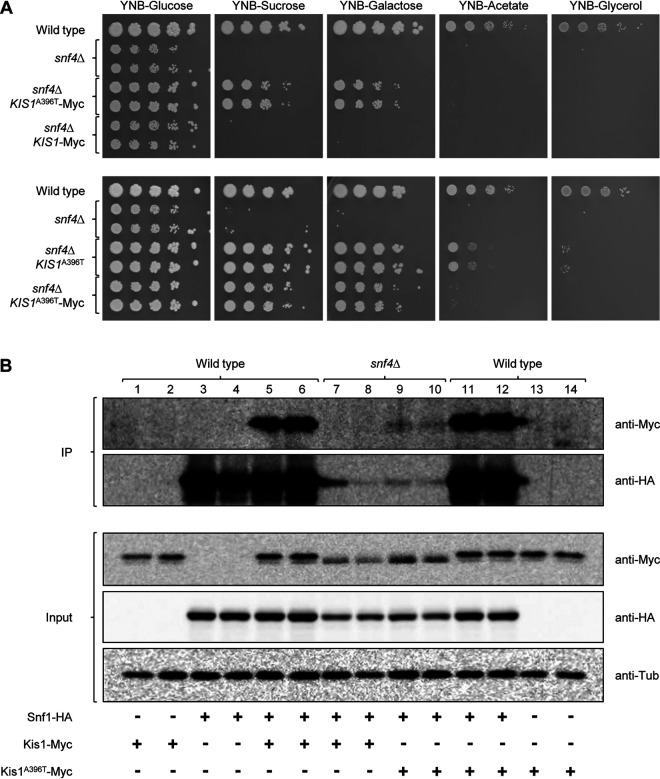
The A396T mutation restores binding of Kis1 to Snf1 in the absence of Snf4. (A) YPD overnight cultures of the wild-type strain SC5314, *snf4*Δ mutants, and derivatives of the *snf4*Δ mutants in which one of the endogenous *KIS1* alleles was replaced by a 3×Myc-tagged wild-type or a 3×Myc-tagged *KIS1*^A396T^ allele were adjusted to an OD_600_ of 2.0, and serial 10-fold dilutions were spotted on YNB agar plates containing 2% glucose, sucrose, galactose, acetate, or glycerol as the carbon source. Plates were incubated for 4 days at 30°C. (B) Wild-type and *snf4*Δ strains with or without HA-tagged Snf1 and Myc-tagged Kis1 or Kis1^A396T^, as indicated, were grown to log phase in YPD at 30°C. Protein extracts were prepared and immunoprecipitated with an anti-HA antibody. Immunoprecipitated Snf1 was detected by Western blotting with an anti-HA antibody, and coimmunoprecipitated Kis1 and Kis1^A396T^ with an anti-Myc antibody (top panels, IP). Tagged proteins in the input samples were detected with anti-HA and anti-Myc antibodies (bottom panels, input). Detection of tubulin with an anti-tubulin antibody served as loading control for the input samples. Both independently generated series of strains were used for the experiments in panels A and B.

In S. cerevisiae, association of the β- and γ-subunits with the catalytic α-subunit to form a stable SNF1 heterotrimer is interdependent; binding of Snf4 to Snf1 was abolished in mutants lacking all three β-subunits, and binding of the β-subunit Gal83 was strongly diminished in the absence of Snf4 (30). We hypothesized that the A396T mutation in Kis1 might enable the protein to bind to Snf1 in the absence of Snf4 and thereby partially restore the activity of the kinase. To investigate this possibility, wild-type and mutated Kis1 were C terminally fused with a 3×Myc epitope and the tagged alleles expressed in wild-type and *snf4*Δ strains with or without a 3×HA-tagged *SNF1* allele. The Myc-tagged wild-type Kis1 did not rescue the growth defect of the *snf4*Δ mutants, demonstrating that the Myc tag did not confer a gain-of-function phenotype (Fig. 2A, top panels). The Myc-tagged Kis1^A396T^ retained its *snf4*Δ suppressor activity, but it restored growth of the *snf4*Δ mutants less efficiently than did untagged Kis1^A396T^, demonstrating that the Myc tag somewhat impeded its functionality (Fig. 2A, bottom panels).

Binding of Kis1 to Snf1 was then tested in coimmunoprecipitation experiments. As shown in Fig. 2B, wild-type Kis1 bound to Snf1 in the wild-type strain (lanes 5 and 6), but not in the *snf4*Δ mutants (lanes 7 and 8), demonstrating that Snf4 is required for β-subunit association with Snf1 also in C. albicans. The Myc-tagged Kis1 was not coimmunoprecipitated by the anti-HA antibody in control strains with untagged Snf1 (lanes 1 and 2), and no signal was observed in strains containing untagged Kis1 (lanes 3 and 4), confirming the specificity of the assay. Importantly, the mutated Kis1^A396T^ regained the ability to bind to Snf1 in the absence of Snf4, albeit at apparently reduced levels compared to the wild type (compare lanes 9 and 10 to lanes 5 and 6). In the wild-type background, Kis1^A396T^ bound as efficiently as wild-type Kis1 to Snf1 (compare lanes 11 and 12 to lanes 5 and 6), and no coimmunoprecipitation of the Myc-tagged Kis1^A396T^ occurred in controls with untagged Snf1 (lanes 13 and 14). Of note, the HA-tagged Snf1 was immunoprecipitated much less efficiently from the *snf4*Δ mutants than from wild-type cells, despite only slightly reduced levels in the input samples (compare lanes 3 to 6 to lanes 7 to 10), indicating that the native HA-tagged Snf1 was less accessible to the anti-HA antibody in the complex lacking Snf4 than in the wild-type complex. This, in turn, suggests that binding of the mutated Kis1^A396T^ to Snf1 in the absence of Snf4 is even better than is apparent from the amount of coimmunoprecipitated protein. The ability of the mutated Kis1 to associate with Snf1 in the absence of Snf4 may therefore have partially restored the functionality of the kinase in *snf4*Δ mutants.

### The A396T mutation restores SNF1 functionality in the absence of Snf4 without improving Snf1 phosphorylation.

**FIG 3:**
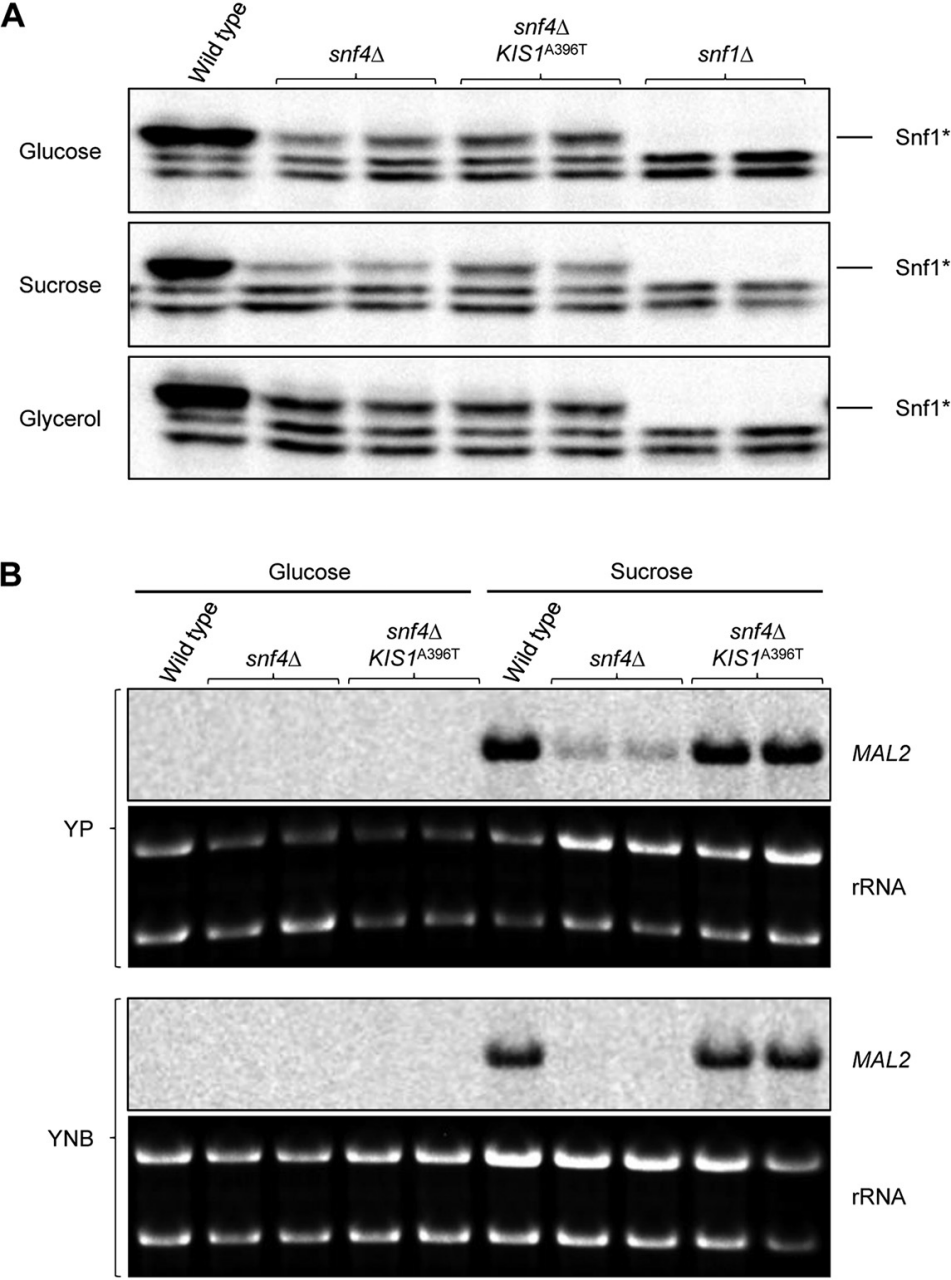
The Kis1^A396T^ mutation restores SNF1 functionality in the absence of Snf4 without improving Snf1 phosphorylation. (A) Thr208 phosphorylation of Snf1 in the wild-type strain SC5314, *snf4*Δ mutants, and *snf4*Δ mutants in which both endogenous *KIS1* alleles were replaced by the *KIS1*^A396T^ allele; *snf1*Δ mutants were included as a negative control. Protein extracts were prepared from cells grown to log phase in YP medium with 2% glucose, sucrose, or glycerol as the main carbon source and analyzed by Western blotting with an antibody against Thr208-phosphorylated Snf1 (Snf1*). The lower bands are from two cross-reacting proteins. (B) Detection of *MAL2* mRNA in the indicated strains grown in YP or YNB media containing 2% glucose or sucrose as carbon source by Northern hybridization; the ethidium bromide-stained gel showing the 25S and 18S rRNAs served as a loading control. Both independently generated series of mutants were used in all experiments.

In C. albicans, Snf1 is constitutively phosphorylated at Thr^208^ by the upstream activating kinase Sak1 (21–23). This phosphorylation is reduced, but not abolished, in *snf4*Δ mutants (21). We speculated that binding of the mutated Kis1^A396T^ to Snf1 might improve Snf1 phosphorylation in the absence of Snf4. However, no increased Snf1 phosphorylation was observed in *snf4*Δ mutants containing two mutated *KIS1*^A396T^ alleles compared to *snf4*Δ mutants containing the wild-type *KIS1* alleles (Fig. 3A). We therefore tested whether the expression of the *MAL2* gene, which is repressed by glucose and induced when sucrose or maltose are used as carbon sources (31), was affected in *snf4*Δ mutants and restored by the Kis1^A396T^ mutation. *MAL2* transcript levels were not detectable by Northern hybridization in wild-type cells grown on glucose, but were strongly upregulated when sucrose was the main (YP-sucrose) or sole (YNB-sucrose) carbon source (Fig. 3B). This induction was strongly diminished or abolished, respectively, in *snf4*Δ mutants and returned to wild-type levels by the mutated Kis1^A396T^. These results indicate that the Kis1^A396T^ mutation restores SNF1 functionality in the absence of Snf4 without improving Snf1 phosphorylation.

### The mutated Kis1^A396T^ enables Mig1 and Mig2 downregulation in *snf4*Δ mutants during growth on alternative carbon sources.

**FIG 4:**
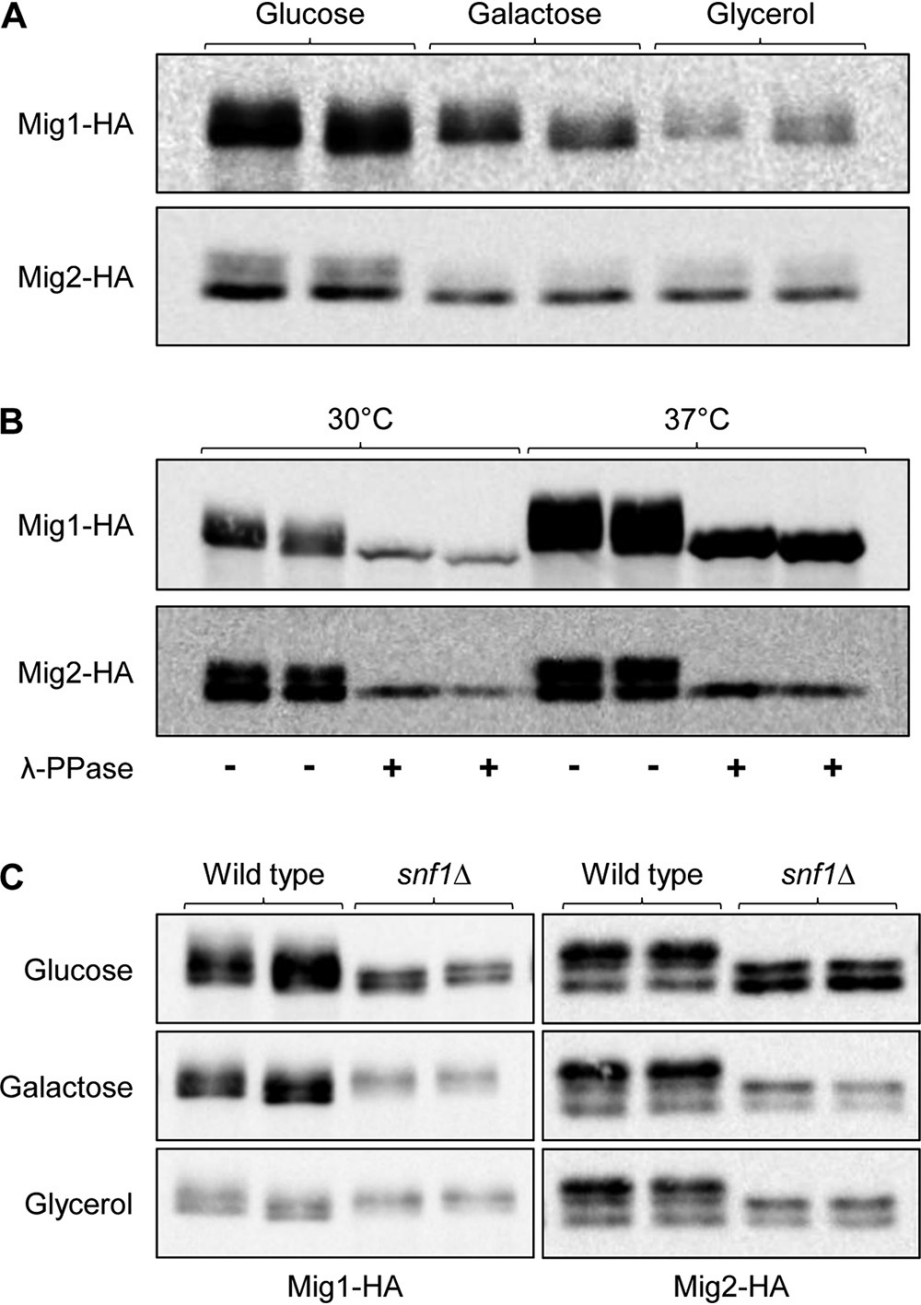
Mig1 and Mig2 are phosphorylated in an Snf1-independent fashion. (A) YPD overnight cultures of the wild-type strain SC5314 containing 3×HA-tagged Mig1 or Mig2 were diluted in fresh YPD medium and grown for 2 h at 30°C. Cells were centrifuged; resuspended in YP medium containing 2% glucose, galactose, or glycerol; and grown for 2 additional hours. Protein extracts were prepared and analyzed by Western blotting with an anti-HA antibody. (B) Overnight cultures of the same strains grown in YPD at 30°C or 37°C were diluted in fresh YPD medium and grown for 4 h at 30 or 37°C. Protein samples were prepared and treated with λ-phosphatase or left untreated and analyzed by Western blotting with an anti-HA antibody. (C) YPD overnight cultures of wild-type and *snf1*Δ strains containing 3×HA-tagged Mig1 or Mig2 were grown at 37°C and treated as described for panel A. Results with both independently generated series of strains are shown in panels A to C.

Since deletion of the repressor-encoding genes *MIG1* and *MIG2* mitigates the growth defects of *snf1*Δ and *snf4*Δ mutants (25, 28), we investigated if the amounts or phosphorylation of Mig1 and Mig2 were affected in the absence of a functional SNF1 complex. To this aim, we replaced one of the endogenous *MIG1* or *MIG2* alleles by a 3×HA-tagged copy in wild-type and *snf1*Δ backgrounds and detected the tagged proteins by Western blotting. For both Mig1 and Mig2, several bands with different mobilities were observed in cells grown with glucose as the main carbon source, and the amounts of these proteins were reduced when glucose was replaced by the alternative carbon sources galactose or glycerol (Fig. 4A; see Fig. S1 for loading controls of all Western blots). Phosphatase treatment of the protein samples resulted in the disappearance of the bands with slower mobility, indicating that these were phosphorylated forms of Mig1 and Mig2 (Fig. 4B). Since *snf1*Δ mutants grow very poorly at 30°C even on glucose but much better at 37°C (25), the latter experiment was also performed with cells grown at 37°C, at which the total amounts of Mig1 and Mig2 and the proportions of their phosphorylated relative to the unphosphorylated forms were increased (Fig. 4B). Interestingly, lower-mobility forms of Mig1 and Mig2 were also detected in *snf1*Δ mutants, regardless of the carbon source (Fig. 4C). These results provide evidence that Mig1 and Mig2 are phosphorylated in an Snf1-independent fashion, but their phosphorylation patterns were altered in the absence of the kinase.

10.1128/msphere.00929-21.1 FIG S1 Loading controls for the Western blots. Download FIG S1, PDF file, 0.5 MB. Copyright © 2021 Ramírez-Zavala et al. 2021 Ramírez-Zavala et al. https://creativecommons.org/licenses/by/4.0/ This content is distributed under the terms of the Creative Commons Attribution 4.0 International license.

**FIG 5:**
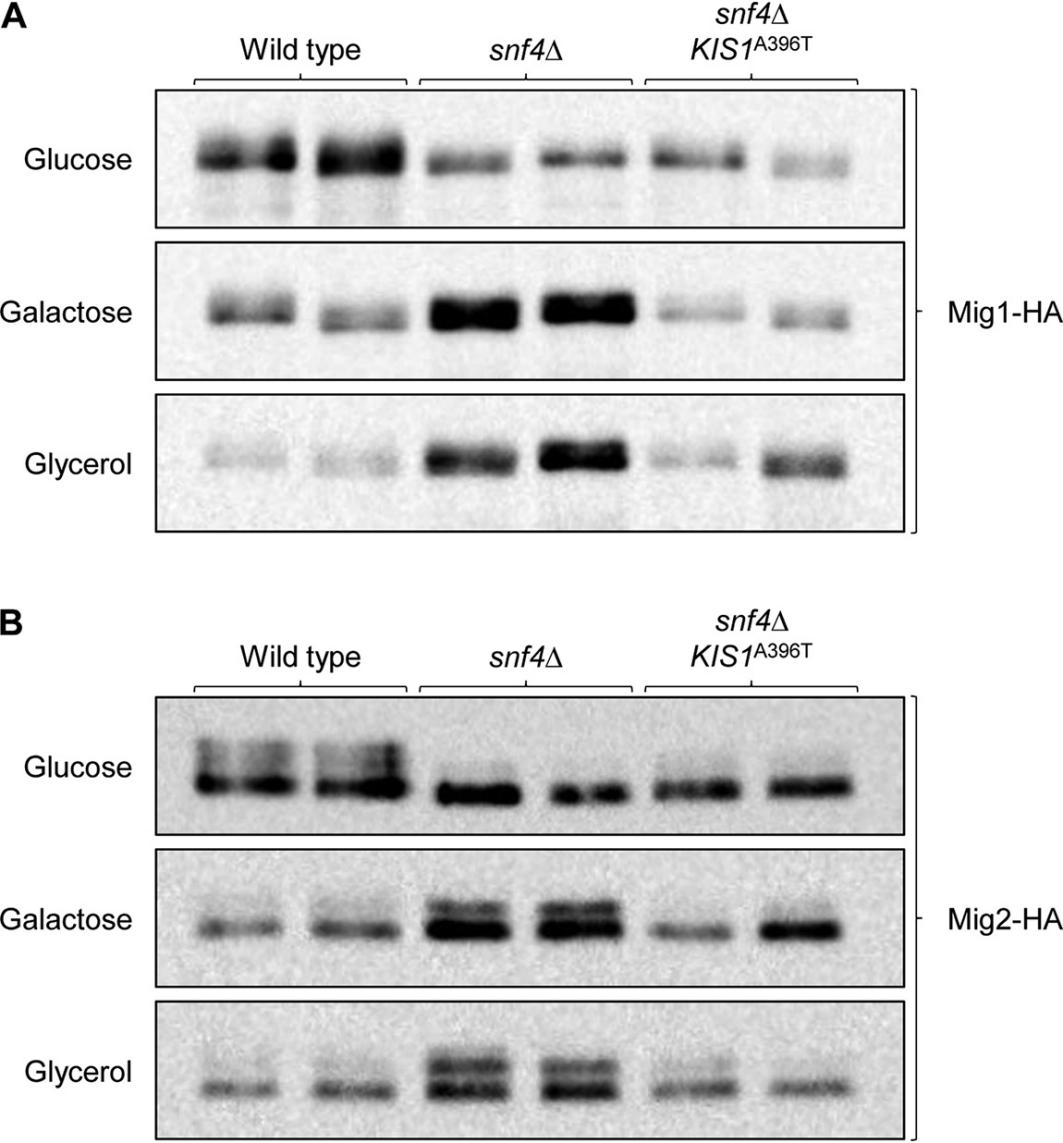
Detection of 3×HA-tagged Mig1 (A) and Mig2 (B) in the wild-type strain SC5314, *snf4*Δ mutants, and *snf4*Δ mutants in which both endogenous *KIS1* alleles were replaced by the *KIS1*^A396T^ allele. YPD overnight cultures of the strains were diluted in fresh YPD medium and grown for 2 h at 30°C. Cells were centrifuged; resuspended in YP medium containing 2% glucose, galactose, or glycerol; and grown for 2 additional hours. Protein extracts were prepared and analyzed by Western blotting with an anti-HA antibody. Results with both independently generated series of strains are shown in each case.

We then tested whether Mig1 and Mig2 were also affected by the absence of the regulatory subunit Snf4 and, in that case, if the mutated Kis1^A396T^ would restore the wild-type situation in *snf4*Δ mutants. Therefore, one of the endogenous *MIG1* or *MIG2* alleles was also 3×HA-tagged in *snf4*Δ mutants and derivatives that were homozygous for the *KIS1*^A396T^ mutation. As can be seen in Fig. 5, the total amounts of phosphorylated and unphosphorylated Mig1 and Mig2 were increased in *snf4*Δ mutants compared to wild-type levels when the cells were grown on the alternative carbon sources galactose or glycerol, and this increase was reverted by the mutated Kis1^A396T^. These results suggest that *snf4*Δ mutants cannot grow on alternative carbon sources because they are unable to reduce the levels of the repressors Mig1 and Mig2. The mutated Kis1^A396T^ sufficiently restores SNF1 functionality in the absence of Snf4 to enable the downregulation of Mig1 and Mig2 levels that is required for growth on carbon sources other than glucose.

## DISCUSSION

Like in other organisms, the regulatory γ-subunit Snf4 is important for the functionality of the SNF1 complex in C. albicans, although the catalytic α-subunit Snf1 has some residual function in the absence of Snf4, as indicated by the more severe growth defect of *snf1*Δ mutants compared to *snf4*Δ mutants on the preferred carbon source glucose (25). Here, we show that SNF1 functionality can be restored to a large extent in the absence of Snf4 by a suppressor mutation in the β-subunit Kis1. Unlike wild-type Kis1, the mutated Kis1^A396T^ could bind Snf1 in the absence of Snf4, which may have relieved Snf1 from autoinhibition, a function that is normally performed by and dependent on Snf4. Although phosphorylation of Snf1 at Thr208 in the activation loop, which is reduced in the absence of Snf4, was not improved, the mutated Kis1^A396T^ enabled the SNF1-dependent induction of the *MAL2* gene, which encodes an α-glucosidase that utilizes sucrose and maltose as the substrates (31). It is likely that the expression of other SNF1-dependent genes and the activity of required enzymes and transporters was also sufficiently restored by the presence of Kis1^A396T^ in the *snf4*Δ mutants to support the observed growth on various alternative carbon sources.

Deletion or mutation of the conserved glycogen-binding domain in the β-subunit Gal83 of the S. cerevisiae SNF1 complex has been reported to render SNF1 constitutively active (32, 33). However, the functionality of SNF1 containing the mutated forms of Gal83 still depended on the presence of Snf4 (33). To our knowledge, the A396T mutation in Kis1 of C. albicans is the first mutation in a β-subunit that renders SNF1 functional in the absence of its γ-subunit. Interestingly, both Gal83 and its paralogue Sip2 contain a threonine at the corresponding position (414 in Gal83 and 410 in Sip2), which is located in another highly conserved sequence at the C terminus of the β-subunits (see Fig. S2). Nevertheless, despite the presence of this threonine in two of its β-subunits, SNF1 activity requires the γ-subunit Snf4 in S. cerevisiae.

10.1128/msphere.00929-21.2 FIG S2 Comparison of the C-terminal sequences of the SNF1 β-subunits Kis1 of C. albicans and Gal83 and Sip2 of S. cerevisiae. Download FIG S2, PDF file, 0.1 MB. Copyright © 2021 Ramírez-Zavala et al. 2021 Ramírez-Zavala et al. https://creativecommons.org/licenses/by/4.0/ This content is distributed under the terms of the Creative Commons Attribution 4.0 International license.

**FIG 6:**
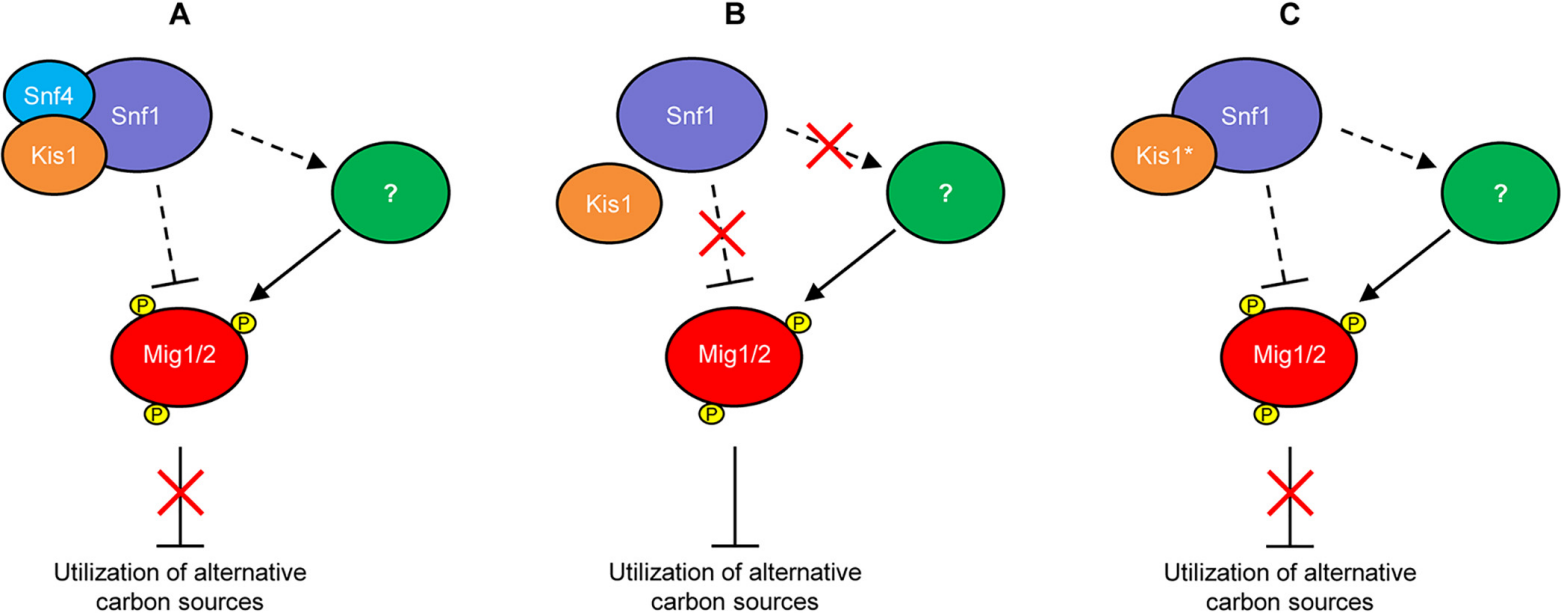
Model illustrating the regulation of Mig1 and Mig2 by the SNF1 complex and additional unknown regulator(s) in C. albicans. (A) In the wild type, SNF1 is required to inhibit the transcriptional repressors Mig1 and Mig2 and enable growth on alternative carbon sources, which may occur by direct phosphorylation and/or effects on other unidentified kinases (symbolized by the question mark) that act on Mig1 and Mig2. (B and C) In contrast to the situation in the model yeast S. cerevisiae, Mig1 and Mig2 are also phosphorylated in the absence of a functional SNF1 complex in C. albicans, demonstrating that additional kinase(s) phosphorylate the repressors. SNF1 functionality is impeded in the absence of the γ-subunit Snf4 (B) but restored by Kis1 containing the A396T suppressor mutation (Kis1*, C). How phosphorylation of Mig1 and Mig2 at different sites in the proteins affects their activity remains to be established.

A still unresolved question is how SNF1 regulates its downstream targets in C. albicans (Fig. 6). The lack of consensus Snf1 phosphorylation sites in CaMig1 suggested that the Snf1 signaling pathways in S. cerevisiae and C. albicans are different (27), but the recent finding that deletion of Mig1 and Mig2 restores growth of *snf1*Δ and *snf4*Δ mutants (25, 28) indicated that the repressors are direct (via alternative phosphorylation sites) or indirect SNF1 downstream targets. In analogy to the situation in S. cerevisiae, we expected that Mig1 and Mig2 would be phosphorylated, and thereby inactivated, in an SNF1-dependent fashion upon growth on alternative carbon sources. However, under our experimental conditions, both Mig1 and Mig2 were partially phosphorylated even in YPD medium, which contains large amounts of glucose as the main carbon source, and the relative proportion of the phosphorylated forms did not increase upon a switch to galactose or glycerol as carbon sources. Instead, the total levels of both proteins decreased under these conditions. This observation confirms and extends the findings by Lagree et al. (28), who also reported that Mig1 levels decreased during growth of C. albicans on alternative carbon sources. Furthermore, we found that Mig1 and Mig2 are phosphorylated even in cells lacking Snf1, although their migration patterns in a protein gel were altered, demonstrating that at least some phosphorylation of the two repressors occurs in an Snf1-independent fashion. The relevance of Mig1 and Mig2 phosphorylation and possibly other protein modifications in the regulation of their activity remains to be established. Importantly, Mig1 and Mig2 levels were higher in *snf4*Δ mutants than in the wild type when glucose was replaced by galactose or glycerol, and they reverted to wild-type levels in the presence of the mutated Kis1^A396T^, providing further evidence that the mutated β-subunit restored SNF1 functionality in the absence of Snf4 by enabling it to downregulate the amounts of the repressors. We note that, counterintuitively, Mig1 and Mig2 levels were generally lower in *snf1*Δ mutants than in the wild type (see Fig. 4C), which could be due to the general growth deficiency of these mutants. The reduced growth of the *snf1*Δ mutants even on glucose may cause a constitutive downregulation of Mig1 and Mig2 by alternative regulatory pathways. Nevertheless, the repressors must still be sufficiently active to inhibit the utilization of alternative carbon sources, since their deletion restores growth of *snf1*Δ mutants (28). Furthermore, downregulation of Mig1 and Mig2 levels was not sufficient to enable utilization of alternative carbon sources in the absence of Snf1, as is evident by the inability of the *snf1*Δ mutants to grow under these conditions (25). In summary, our results provide novel insights into the regulation of SNF1 and the repressors Mig1 and Mig2 in the metabolic adaptation of C. albicans, and they point out open questions for future research, such as which other regulators control Mig1 and Mig2 activity and how these are affected by SNF1.

## MATERIALS AND METHODS

### Strains and growth conditions.

The C. albicans strains used in this study are listed in Table S1 in the supplemental material. All strains were stored as frozen stocks with 17.2% glycerol at −80°C and subcultured on YPD agar plates (10 g of yeast extract, 20 g of peptone, 20 g of glucose, and 15 g of agar per L) at 30°C. Strains were routinely grown in YPD liquid medium at 30°C in a shaking incubator. Exceptions were *snf1*Δ mutants, which were grown at 37°C on solid and in liquid media because they grow very poorly at 30°C (25). For selection of transformants, 200 μg/mL nourseothricin (Werner Bioagents) or 1 mg/mL hygromycin B was added to YPD agar plates. To obtain nourseothricin-sensitive derivatives in which the *SAT1* flipper cassette was excised by FLP-mediated recombination, transformants were grown overnight in YCB-BSA-YE medium (23.4 g of yeast carbon base, 4 g of bovine serum albumin, and 2 g yeast extract per L [pH 4.0]) without selective pressure to induce the *SAP2* promoter controlling *caFLP* expression. Appropriate dilutions were plated on YPD agar plates and grown for 2 days at 30°C. Individual colonies were picked and streaked on YPD plates and on YPD plates with 100 μg/mL nourseothricin to confirm sensitivity.

10.1128/msphere.00929-21.3 TABLE S1 C. albicans strains used in this study. Download Table S1, XLSX file, 0.02 MB. Copyright © 2021 Ramírez-Zavala et al. 2021 Ramírez-Zavala et al. https://creativecommons.org/licenses/by/4.0/ This content is distributed under the terms of the Creative Commons Attribution 4.0 International license.

### Plasmid constructions.

Plasmid pKIS1^A396T^, which contains the G1186A nucleotide substitution in *KIS1*, was generated as follows. A part of the *KIS1* coding region (positions +665 to +1198) was amplified from genomic DNA of strain SC5314 with primers KIS1A396Tfwd and KIS1A396T-N; the latter changes the alanine codon GCA (positions +1186 to +1188 in *KIS1*) into the threonine codon ACA. A fragment comprising the *KIS1* coding region from position +1172 and downstream sequences was amplified with primers KIS1A396T-C (complementary to KIS1A396T-N) and KIS1A396Trev. The PCR products served as the templates in a subsequent fusion PCR with primers KIS1A396Tfwd and KIS1A396Trev. The fused fragment was digested at the introduced SacI and SacII sites and substituted for the 5′ *KIS1* flanking sequence in the *KIS1* deletion cassette contained in plasmid pKIS1M1 (21). To generate 3×Myc-tagged versions of wild-type *KIS1* and *KIS1*^A396T^, the *KIS1* coding and upstream sequences were amplified from the heterozygous strain SCΔ*snf4*KIS1^A396T^M1A, which contains both wild-type and mutant alleles, with primers KIS1.01 and KIS1.06; the latter primer introduces a BamHI site, encoding a Gly-Ser linker, instead of the *KIS1* stop codon. A fragment encoding three copies of the Myc epitope, followed by a stop codon and the transcription termination sequence of the *ACT1* gene, was amplified from pCEK1H1 (34) with primers 3×Myc-ACT1T and ACT19. The PCR products were digested with SacI/BamHI and BamHI/SacII, respectively, and inserted together in the SacI/SacII-digested pKIS1M1. The resulting plasmids pKIS1Myc3 and pKIS1^A396T^Myc3 contain the 3×Myc-tagged wild-type *KIS1* and *KIS1*^A396T^ alleles, respectively. To construct a 3×HA-tagged *MIG1* allele, a fragment encoding the C-terminal region of Mig1 was amplified with primers MIG1HAfwd and MIG1HArev; the latter primer introduces a BamHI site instead of the *MIG1* stop codon. The PCR product was digested with SacI and BamHI and inserted together with a BamHI-SacII fragment from pSNF1H1 encoding three copies of the HA epitope, followed by a stop codon and the transcription termination sequence of the *ACT1* gene, instead of the 5′ *MIG1* flanking sequence in the *MIG1* deletion cassette contained in plasmid pMIG1M1 (25) to obtain pMIG1H1. A 3×HA-tagged *MIG2* allele was generated in an analogous fashion using primers MIG2HAfwd and MIG2HArev, yielding pMIG2H1. In plasmids pMIG1H2 and pMIG2H2, the *caSAT1* marker of pMIG1H1 and pMIG2H1, respectively, was replaced by the *HygB* marker from plasmid pSNF1ex3 (25). The sequences of the oligonucleotide primers that were used for plasmid constructions are given in Table S2.

10.1128/msphere.00929-21.4 TABLE S2 Oligonucleotide primers used in this study. Download Table S2, XLSX file, 0.01 MB. Copyright © 2021 Ramírez-Zavala et al. 2021 Ramírez-Zavala et al. https://creativecommons.org/licenses/by/4.0/ This content is distributed under the terms of the Creative Commons Attribution 4.0 International license.

### Strain constructions.

C. albicans strains were transformed by electroporation (35) with the gel-purified inserts from the plasmids described above. The insert from pKIS1^A396T^ was used to sequentially replace the *KIS1* wild-type alleles in the *snf4*Δ mutants SCKIS1M4A and SCKIS1M4B by the mutated *KIS1*^A396T^ allele with the help of the recyclable *SAT1* flipper cassette. The inserts from pKIS1Myc3 and pKIS1^A396T^Myc3 were used to replace one of the endogenous *KIS1* alleles in the wild-type strain SC5314, *snf4*Δ mutants, and derivatives carrying a 3×HA-tagged *SNF1* allele by the 3×Myc-tagged wild-type or mutated *KIS1* alleles. The inserts from pMIG1H1 and pMIG2H1 were used to add a 3×HA tag to one of the endogenous *MIG1* or *MIG2* alleles, respectively, in the wild type, the *snf4*Δ mutants, and derivatives containing mutated *KIS1* alleles. The inserts from pMIG1H2 and pMIG2H2, which contain the hygromycin resistance marker *HygB*, were used for the same purpose in the *snf1*Δ mutants SCSNF1M8A and SCSNF1M8B, which already contain the *caSAT1* marker (25). The correct genomic integration of all constructs and subsequent excision of the *SAT1* flipper cassette were confirmed by Southern hybridization using the flanking sequences as probes.

### Isolation of genomic DNA and Southern hybridization.

Genomic DNA from C. albicans strains was isolated as described previously (36). The DNA was digested with appropriate restriction enzymes, separated on a 1% agarose gel, transferred by vacuum blotting onto a nylon membrane, and fixed by UV cross-linking. Southern hybridization with enhanced chemiluminescence-labeled probes was performed with the Amersham ECL Direct Nucleic Acid Labeling and Detection System (Cytiva) according to the instructions of the manufacturer.

### Growth assays.

To test growth on different carbon sources, YPD overnight cultures of the strains were adjusted to an optical density at 600 nm (OD_600_) of 2.0 in water, serially 10-fold diluted, and spotted on YP (1% yeast extract, 2% peptone, 1.5% agar) or YNB (0.67% yeast nitrogen base with ammonium sulfate, 2% agar) plates containing 2% glucose, sucrose, galactose, potassium acetate, or glycerol as carbon source. Plates were incubated for 2 or 4 days at 30°C.

### Immunoprecipitation experiments.

Overnight cultures of the strains were diluted 10^−2^ in fresh YPD medium and grown for 4 h at 30°C. Cells were collected by centrifugation, washed with ice-cold water, and resuspended in 300 μL of breaking buffer (50 mM Tris-HCl [pH 8], 250 mM NaCl, 5 mM EDTA, 0.1% [vol/vol] Triton X-100, cOmplete EDTA-free protease inhibitor cocktail and PhosStop phosphatase inhibitor cocktail [Roche]). An equal volume of 0.5 mm acid-washed glass beads was added to each tube. Cells were mechanically disrupted on a FastPrep-24 cell-homogenizer (MP Biomedicals) with three 40-s pulses, with 5 min on ice between each pulse. Cell lysates were centrifuged at 21,000 × *g* for 20 min at 4°C, the supernatant was collected, and the protein concentration was quantified using the Bradford protein assay. Equal amounts of total protein were immunoprecipitated with 30 μL of anti-HA agarose resin (Pierce Biotechnology) overnight at 4°C. The resin was washed three times with TBST (Tris-buffered saline with 0.05% Tween 20), and bound proteins were eluted with 3 M NaSCN. Eluted samples were desalted using Amicon Ultra 30K devices (Merck). All immunoprecipitated samples and corresponding input samples were resolved on SDS–10% polyacrylamide gels and analyzed by Western blotting (see below).

### Western blotting.

Strains were grown in YP media containing different carbon sources as detailed in the legends to the figures. Cell lysates were prepared as described above for the immunoprecipitation experiments, except that centrifugation was performed for 15 min at 21,000 × *g*. Equal amounts of protein of each sample were mixed with one volume of 2× Laemmli buffer, heated for 5 min at 95°C, and separated on an SDS–8% polyacrylamide gel. Separated proteins were transferred onto a nitrocellulose membrane with a mini-Protean System (Bio-Rad) and stained with Ponceau S to control for equal loading (see Fig. S1 for all loading controls). To detect T208-phosphorylated Snf1, the membrane was blocked with 5% BSA in TBST and subsequently incubated overnight at 4°C with Phospho-AMPKα (Thr172) antibody 2531 (Cell Signaling Technology). The membrane was washed in TBST and incubated at room temperature for 1 h with anti-rabbit HRP G-21234 antibody (Invitrogen). For the detection of HA-tagged proteins, membranes were blocked with 5% milk in TBST and incubated overnight with rat monoclonal anti-HA-peroxidase antibody, clone 3F10 (Roche). For the detection of Myc-tagged proteins, membranes were blocked with 5% milk in TBST and incubated overnight with anti-Myc (71D10) Rabbit MAb 2278 (Cell Signaling Technology). Membranes were washed with TBST and then incubated with anti-rabbit HRP G-21234 antibody. To detect tubulin, membranes were blocked with 5% milk in TBST and incubated overnight at 4°C with rat anti-tubulin alpha antibody MCA 78G (Bio-Rad), washed with TBST, and then incubated with rabbit anti-rat HRP-conjugated antibody STAR21B (Bio-Rad). To reprobe the immunoblots, membranes were incubated in stripping buffer (0.2 M glycine, 0.1% SDS, 1% Tween 20 [pH 2.2]) and washed in PBS and TBST before blocking with 5% milk. Signals were generated with the ECL chemiluminescence detection system (Cytiva) and captured with the ImageQuant LAS 4000 imaging system (Cytiva). For phosphatase treatment, the cell extracts were prepared as described above, except that the phosphatase inhibitor cocktail was omitted in the breaking buffer. Cell extracts were incubated with λ protein phosphatase (New England Biolabs) at 30°C for 30 min.

### Northern hybridization.

YPD overnight cultures of the strains were diluted 10^−2^ in YP media containing 2% glucose or sucrose and grown for 4 h at 30°C. Total RNA was extracted using a Quick-RNA fungal/bacterial miniprep kit (Zymo Research) according to the manufacturer’s instructions. RNA samples were separated on a 1.2% agarose gel, stained with ethidium bromide, transferred by capillary blotting onto a nylon membrane, and fixed by UV cross-linking. The blot was hybridized with a digoxigenin-labeled *MAL2*-specific probe (positions +1322 to +1625 in the coding sequence), which was amplified with the primers MAL2NBFw and MAL2NBRv (see Table S2). Bound probe was detected with a peroxidase-labeled anti-digoxigenin AP-conjugate (Roche). Signals were generated using CSPD (Roche) as the substrate and captured with the ImageQuant LAS 4000 imaging system.
