# Supplementary material for: A Suppressor Mutation in the β-Subunit Kis1 Restores Functionality of the SNF1 Complex in Candida albicans snf4Δ Mutants
Source: mSphere. 2021 Dec 15;6(6):e00929-21. doi: 10.1128/msphere.00929-21 (PMC8673253; doi:10.1128/msphere.00929-21)

Loading control for Fig. 2B (Ponceau S-stained input samples)

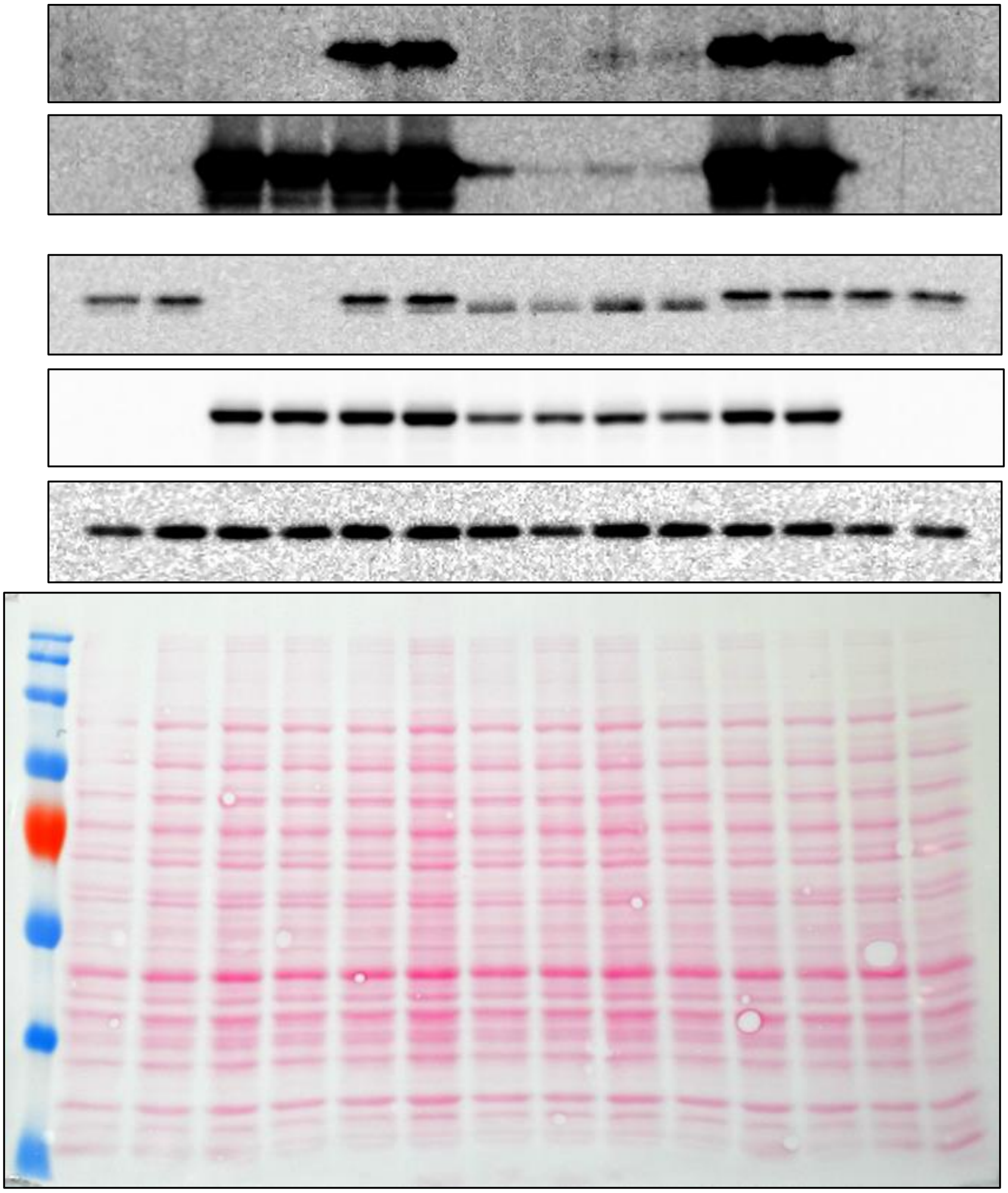

FIG S1 Loading controls for the Western blots (continued on the next pages).

Loading controls for Fig. 3A (Ponceau S-staining)

Glucose

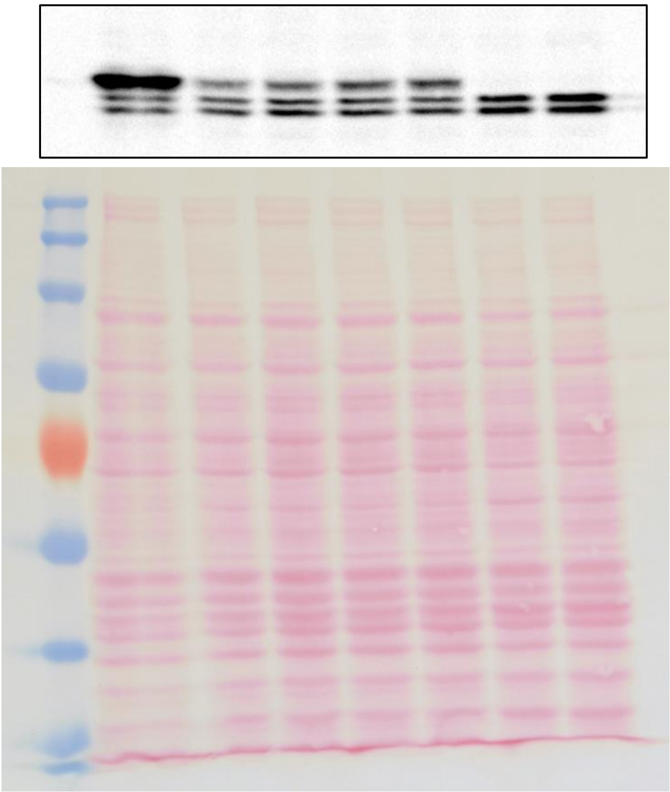

Glycerol | Sucrose

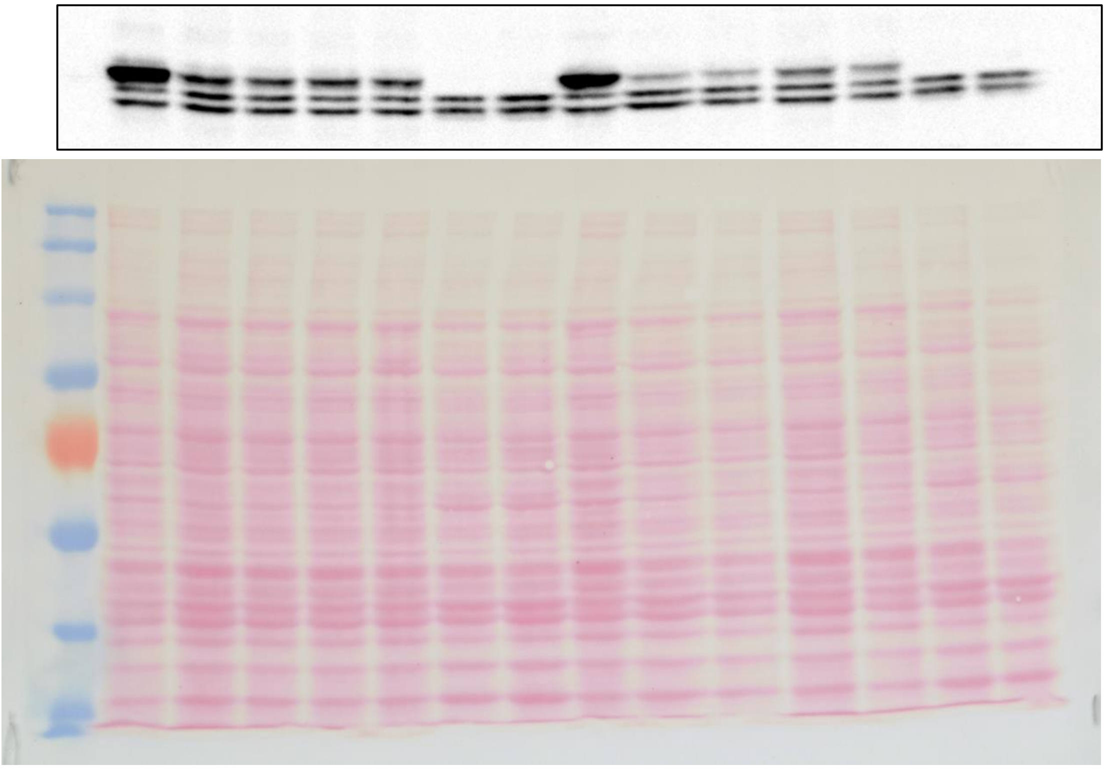

Loading controls for Fig. 4A (Ponceau S-staining)

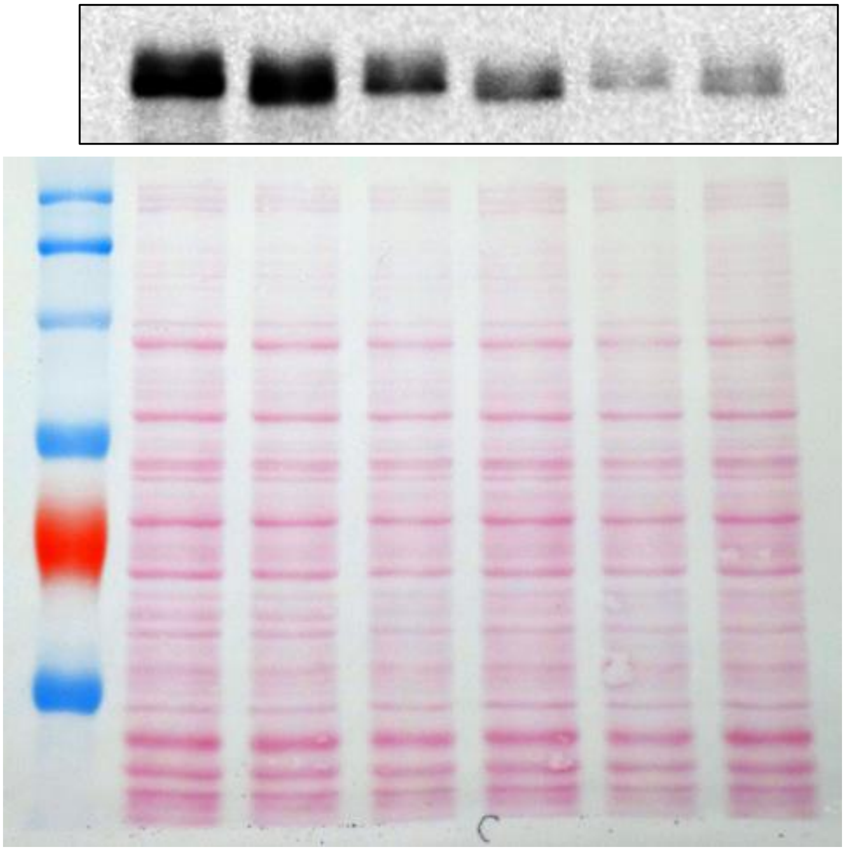

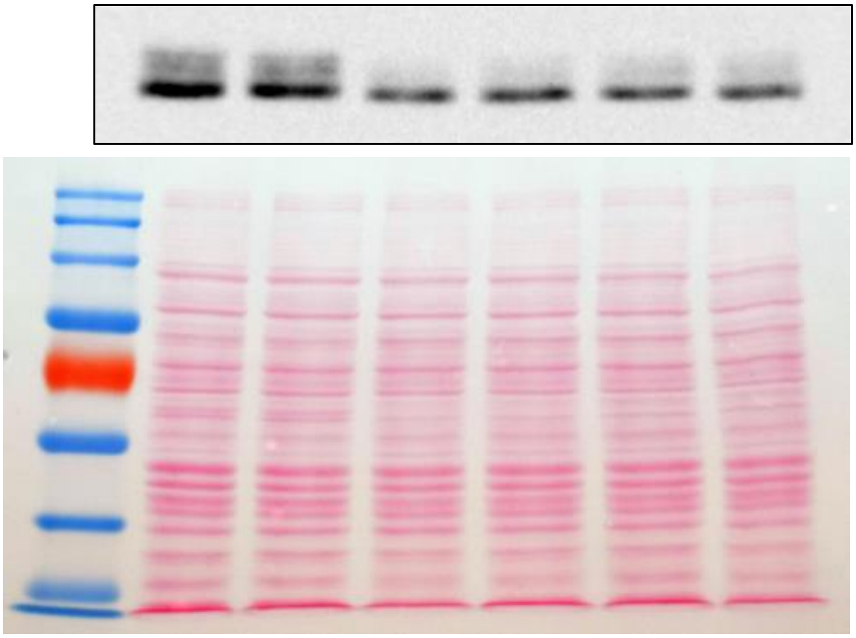

Loading controls for Fig. 4B (Ponceau S-staining)

Mig1-HA

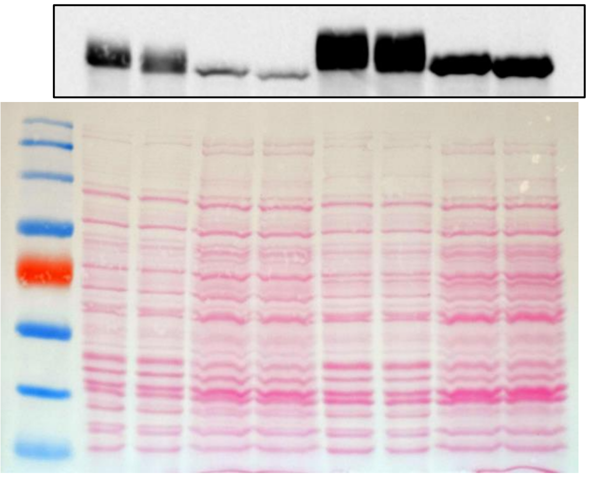

Mig2-HA

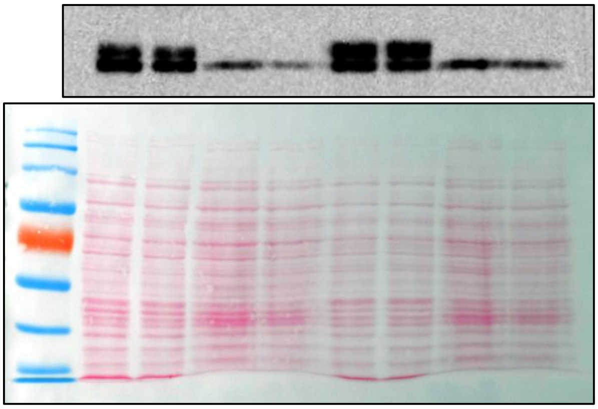

Loading controls for Fig. 4C (Ponceau S-staining)

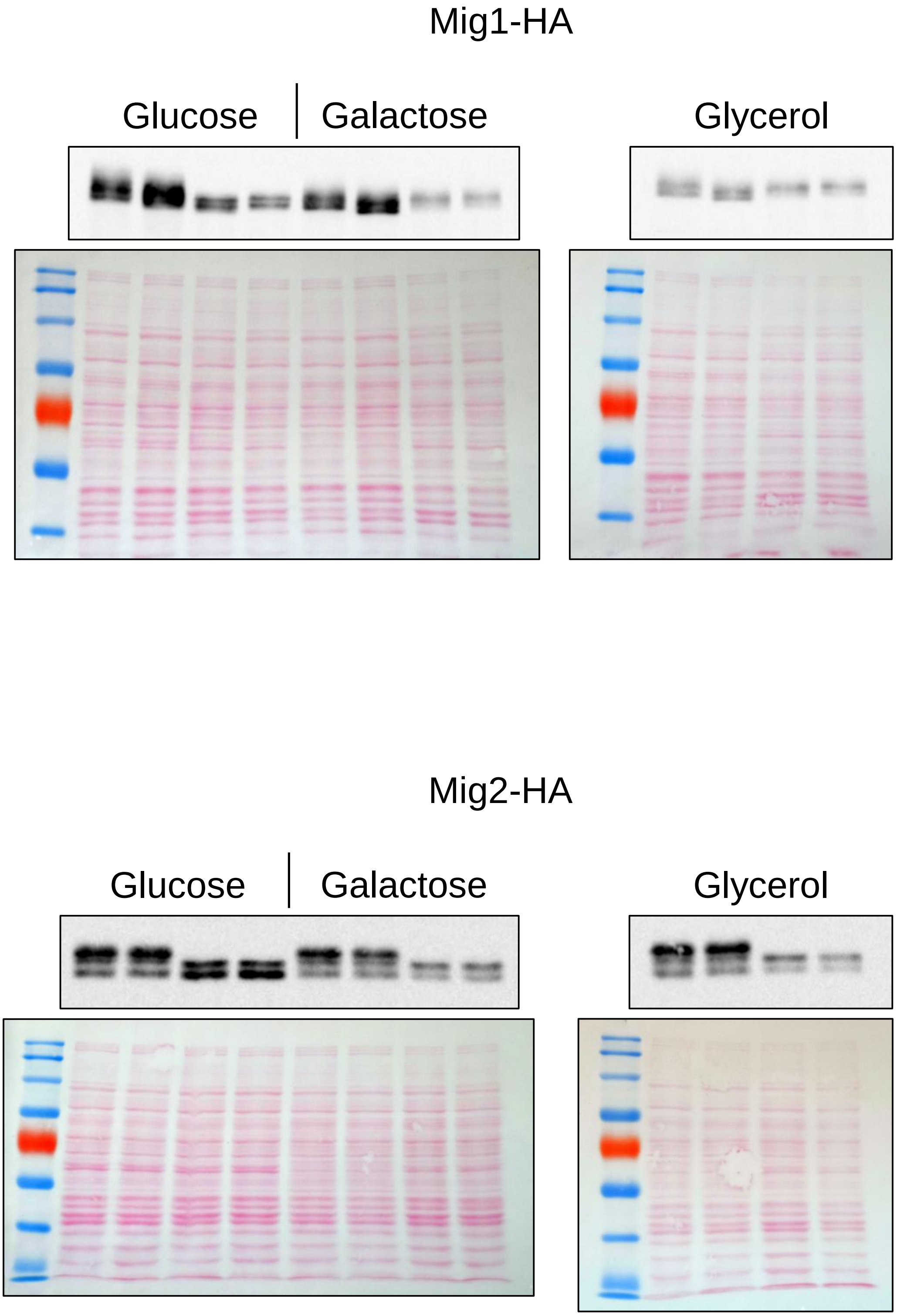

Loading controls for Fig. 5A (Ponceau S-staining)

Glucose

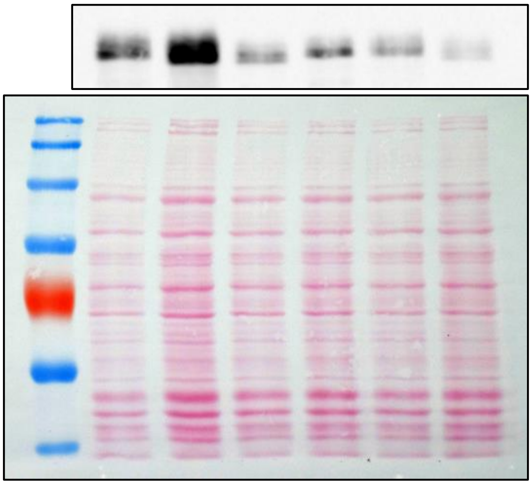

Galactose

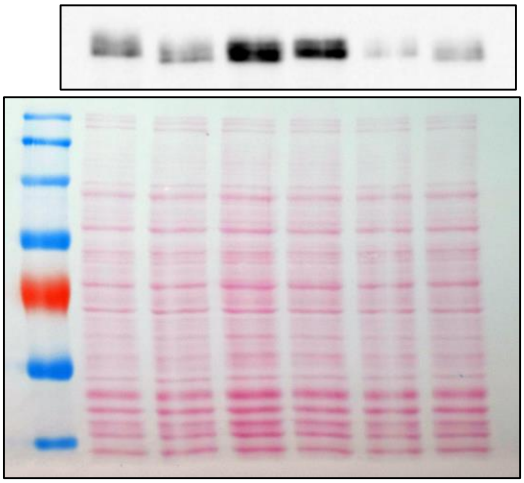

Glycerol

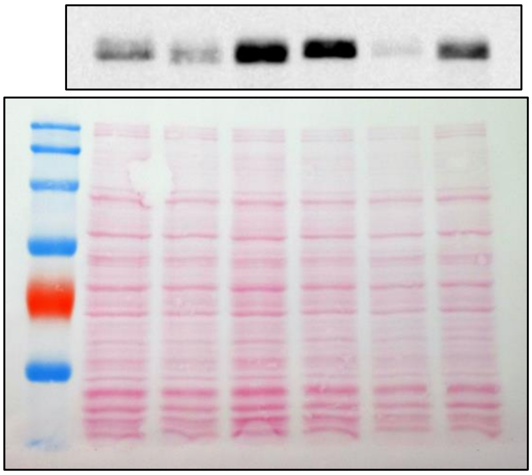

Loading controls for Fig. 5B (Ponceau S-staining)

Glucose

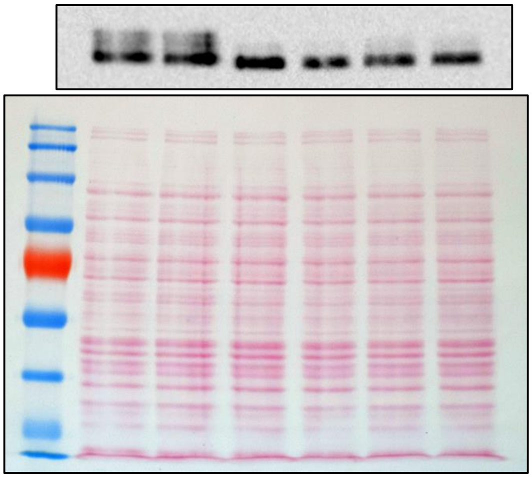

Galactose

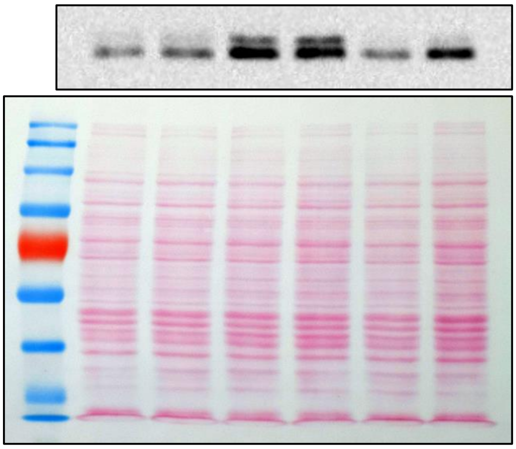

Glycerol

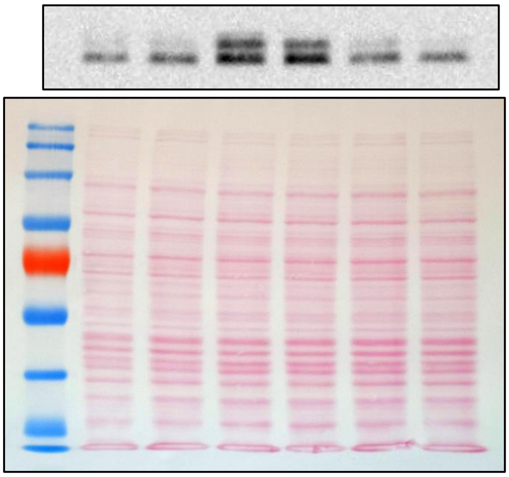

Supplement: FIG S1 [file msphere.00929-21-sf001.pdf]
