# Supplementary material for: A Suppressor Mutation in the β-Subunit Kis1 Restores Functionality of the SNF1 Complex in Candida albicans snf4Δ Mutants
Source: mSphere. 2021 Dec 15;6(6):e00929-21. doi: 10.1128/msphere.00929-21 (PMC8673253; doi:10.1128/msphere.00929-21)
Supplement: FIG S2 [file msphere.00929-21-sf002.pdf]

```
379 VASIVRYKRKYLTQVLYAPLQQASSSSTTQEQQS* 412 Kis1
397 VASIVRYKQKYVTQILYTPLQ*              417 Gal83
393 VASIVRYKQKYVTQILYTPIESS*           415 Sip2
```

FIG S2 Comparison of the C-terminal sequences of the SNF1 β-subunits Kis1 of *C. albicans* and Gal83 and Sip2 of *S. cerevisiae*. Amino acid residues that are identical in all three proteins are shown in green, and the alanine at position 396 of Kis1, which is mutated to threonine in the *snf4*Δ suppressor mutant Kis1^{A396T}, is highlighted in red.
